# Lifetime experience of multiple common mental disorders and 19-year mortality: results from a Canadian population-based cohort

**DOI:** 10.1017/S2045796018000859

**Published:** 2019-02-04

**Authors:** M. Kingsbury, E. Sucha, N. J. Horton, H. Sampasa-Kanyinga, J. M. Murphy, S. E. Gilman, I. Colman

**Affiliations:** 1 School of Epidemiology and Public Health, University of Ottawa, Ottawa, ON, Canada; 2 Department of Mathematics and Statistics, Amherst College, Amherst, MA, USA; 3 Department of Psychiatry, Massachusetts General Hospital and Harvard Medical School, Boston, MA, USA; 4 Department of Epidemiology, Harvard TH Chan School of Public Health, Boston, MA, USA; 5 Department of Psychiatry, Dalhousie University Faculty of Medicine, Halifax, NS, Canada; 6 Social and Behavioral Sciences Branch, Division of Intramural Population Health Research, Eunice Kennedy Shriver National Institute of Child Health and Human Development, Bethesda, MD, USA; 7 Department of Mental Health, Johns Hopkins Bloomberg School of Public Health, Baltimore, MD, USA

**Keywords:** Alcohol abuse, common mental disorders, epidemiology, health outcomes, multimorbidity

## Abstract

**Aims:**

To examine the impact of multiple psychiatric disorders over the lifetime on risk of mortality in the general population.

**Methods:**

Data came from a random community-based sample of 1397 adults in Atlantic Canada, recruited in 1992. Major depression, dysthymia, panic disorder, generalised anxiety disorder and alcohol use disorders were assessed using the Diagnostic Interview Schedule (DIS). Vital status of participants through 2011 was determined using probabilistic linkages to the Canadian Mortality Database. Cox proportional hazard models with age at study entry as the time scale were used to investigate the relationship between DIS diagnoses and mortality, adjusted for participant education, smoking and obesity at baseline.

**Results:**

Results suggested that mood and anxiety disorders rarely presented in isolation – the majority of participants experienced multiple psychiatric disorders over the lifetime. Elevated risk of death was found among men with both major depression and dysthymia (HR 2.56; 95% CI 1.12–5.89), depression and alcohol use disorders (HR 2.45; 95% CI 1.18–5.10) and among men and women who experienced both panic disorder and alcohol use disorders (HR 3.80; 95% CI 1.19–12.16).

**Conclusion:**

The experience of multiple mental disorders over the lifetime is extremely common, and associated with increased risk of mortality, most notably among men. Clinicians should be aware of the importance of considering contemporaneous symptoms of multiple psychiatric conditions.

Common mental disorders are a leading contributor to the global burden of disease (Prince *et al*., 2007; Whiteford *et al*., 2013), and are associated with excess mortality (Walker *et al*., 2015). In particular, major depressive disorder (MDD) has been consistently associated with higher risk of mortality (Cuijpers *et al*., 2014*a*), most strongly among men (Cuijpers *et al*., 2014*b*; Gilman *et al*., 2017; Colman *et al*., 2018). There is less consensus on the relationship between anxiety disorders and mortality. Results from some studies have suggested that anxiety disorders, like depression, are associated with excess mortality (van Hout *et al*., 2004; Kikkenborg Berg *et al*., 2014). Others have suggested that anxiety may be protective against accidental mortality in early adulthood, but confer increased risk of non-accidental mortality later in life (Lee *et al*., 2006). Notably, many studies of the relation between anxiety and mortality do not discriminate between specific anxiety disorders, considering generalised anxiety disorder (GAD), panic disorder, obsessive compulsive disorder and others together in a single category (van Hout *et al*., 2004; Laan *et al*., 2011). Others consider ‘trait anxiety’ (Lee *et al*., 2006), or non-specific symptoms of anxiety (Kikkenborg Berg *et al*., 2014).

Psychiatric comorbidity can be defined as the occurrence of two or more mental disorders in the same individual, either concurrently or over the life span (Brown *et al*., 2001). Comorbidity among mental disorders, both concurrently and over the lifetime, is very common (Boyd *et al*., 1984; Brown *et al*., 2001; Kessler *et al*., 2005; Roca *et al*., 2009). In fact, 45–55% of those with a current mood or anxiety disorder meet criteria for at least one other disorder concurrently (Brown *et al*., 2001; Kessler *et al*., 2005), and up to 81% have had at least one additional disorder over their lifetime (Brown *et al*., 2001). The experience of multiple psychiatric disorders over the lifetime is associated with worse functional and health outcomes than are unique diagnoses. For example, lifetime comorbidity with an additional mental disorder is associated with poorer outcomes of mood (Brown *et al*., 1996) and anxiety disorders (Roy-Byrne *et al*., 2000), including greater severity of symptoms (Roy-Byrne *et al*., 2000), higher likelihood of relapse (Brown *et al*., 1996; Roy-Byrne *et al*., 2000) and higher risk of suicidal behaviour (Henriksson *et al*., 1993; Nock *et al*., 2010). Alcohol has been causally linked with over 60 unique medical conditions (Rehm *et al*., 2003), and alcohol use disorders have been associated with numerous deleterious health consequences (Room *et al*., 2005), including increased risk of death due to both natural and unnatural causes (Harris and Barraclough, 1998). When individuals suffer from mood and anxiety disorders in conjunction with alcohol use disorders, there is a substantially higher risk of impairment, disability and suicide (Merikangas *et al*., 1998).

Most studies reporting elevated mortality risk among individuals with psychiatric disorders have focused on risk associated with single disorders (Harris and Barraclough, 1998); there is much less evidence addressing the concern that the experience of multiple disorders further diminishes life expectancy. Laan *et al.* reported that among patients receiving psychiatric services, both anxiety disorders and depression were associated with excess mortality, but that the superimposition of the two disorders did not further inflate risk (Laan *et al*., 2011). Similarly, Naicker *et al.*, using data from a Norwegian population-based study, recently reported that the experience of both depression and anxiety was associated with an increased risk of death – at a level similar to the risk conferred by either disorder alone (Naicker *et al*., 2017). Results from the first wave of the Stirling County study suggested that although depression was associated with excess mortality, the superimposition of anxiety (Murphy *et al*., 1987) or alcohol abuse (Murphy *et al*., 2008) on depression did not confer additional risk. By contrast, Phillips *et al*. reported that among veterans, the presence of comorbid GAD and MDD was associated with a higher risk of mortality than either disorder in isolation (Phillips *et al*., 2009).

## Aims of the study

There is conflicting evidence regarding the extent to which the cumulative experience of multiple psychiatric disorders over the lifetime is itself a risk factor for mortality – beyond the well-established mortality risk associated with individual psychiatric disorders. The purpose of the present study was therefore to evaluate the long-term risk of mortality associated with the comorbidity between pairs of common psychiatric disorder over the lifetime among individuals in the Stirling County Study, a landmark study in psychiatric epidemiology.

## METHODS

### Data source and procedure

The Stirling County Study was established to describe the epidemiology of psychiatric disorders in a community located in Atlantic Canada. Three random and independent samples of the adult population were selected in 1952 (*n* = 1003), 1970 (*n* = 1203) and 1992 (*n* = 1402). Participation rates were consistently above 79% over the course of the study. Participants gave their informed consent to be interviewed in their homes using a structured interview schedule. The current results are based on respondents from the 1992 sample, randomly selected from the county census, who provided sufficient information on psychiatric disorders (*n* = 1397). Ethics approval for the present study was obtained from the Institutional Review Boards of the Massachusetts General Hospital and the Ottawa Health Science Network Research Ethics Board.

### Mental disorders

Mental disorder was assessed at study entry using the Diagnostic Interview Schedule (DIS), a fully structured interview schedule developed for the National Institute of Mental Health (NIMH) Epidemiologic Catchment Area (ECA) Program to implement the diagnostic guidelines of the Third Diagnostic and Statistical Manual (DSM-III) of the American Psychiatric Association (Robins *et al*., 1981). When compared with evaluation by a psychiatrist, the DIS was found to have 93% sensitivity and 83% specificity for the detection of psychiatric disorders (Murphy, 1994). Five common DSM-based diagnoses were available: major depression, dysthymia, panic disorder, GAD and alcohol use disorder.

All DIS diagnoses were made on a lifetime basis. For the present study, alcohol abuse and alcohol dependence were combined into a single indicator of alcohol use disorder.

### Mortality

In order to investigate mortality outcomes for the Stirling County Study population, a probabilistic matching of study participants to the Canadian Mortality Database (CMDB) was performed at Statistics Canada using a Generalized Iterative Record Linkage System (Howe and Lindsay, 1981; Smith and Newcombe, 1982). This process generates probability weights for each potential match between records in the study cohort and the CMDB. Matches with high weights were retained, low weights were discarded and remaining matches were resolved manually. Mortality information was available through 31 December 2011.

### Covariates

In addition to DIS diagnoses, we included several covariates, assessed in 1992, known to be associated with both mental illness and mortality: education (<5^th^, 5^th^–10^th^, 11^th^ grade or higher) (Miech and Shanahan, 2000; Hummer and Hernandez, 2013), smoking (non-smoker, smokes 1–19 cigarettes per day, smokes 20 or more cigarettes per day) (Lasser *et al*., 2000; Ezzati and Lopez, 2003) and obesity (defined by a body mass index above 30) (Adams *et al*., 2006; Luppino *et al*., 2010).

### Statistical analysis

Sex-specific Cox proportional hazard models with age at study entry as the time scale were used to investigate the relationship between DIS diagnoses and mortality in the 1992 sample. Proportionality assumptions of Cox models were checked by including a time-dependent covariate, i.e. an interaction term between the predictor and a function of survival time, in the models and assessing its significance.

The first analysis investigated the association between single disorders and mortality. Individuals in those analyses could have the given disorder with or without comorbid disorders. The reference category for each model consisted of those who did not meet criteria for the index disorder. For example, all individuals with depression were compared with all individuals without depression, regardless of the presence of any comorbid conditions (Table 1). In a sensitivity analysis, participants meeting criteria for a single disorder were compared with those with no disorder (Table 2).

**Table 1.** HRs (95% CIs) for all-cause mortality associated with DIS-based diagnoses of mental disorders; SCS, 1992−2011

				HR (95% CI)					
	No. (%) diagnosed			Adjusted for age			All covariates^b^		
	All (*n* = 1397)	Women (*n* = 759)	Men (*n* = 638)	All^a^	Women	Men	All¹	Women	Men
Depression	114 (8.16%)	83 (10.94%)	31 (4.86%)	1.35 (0.91–2.01)	1.04 (0.61–1.76)	2.12 (1.18–3.82)	1.33 (0.89–1.98)	1.02 (0.60–1.73)	2.08 (1.15–3.75)
Dysthymia	94 (6.73%)	71 (9.35%)	23 (3.61%)	1.22 (0.83–1.79)	1.02 (0.64–1.63)	1.86 (0.99–3.52)	1.15 (0.78–1.68)	0.98 (0.61–1.58)	1.64 (0.85–3.14)
Panic disorder	35 (2.51%)	26 (3.43%)	9 (1.41%)	2.42 (1.35–4.33)	2.19 (1.02–4.68)	2.84 (1.16–6.95)	2.34 (1.30–4.22)	2.07 (0.96–4.45)	2.87 (1.17–7.07)
Gad	280 (20.04%)	177 (23.32%)	108 (16.93%)	1.09 (0.85–1.38)	0.98 (0.70–1.36)	1.23 (0.87–1.74)	1.05 (0.82–1.34)	0.95 (0.68–1.32)	1.18 (0.83–1.70)
Alcohol use disorder	301 (21.56%)	54 (7.12%)	247 (38.71%)	1.21 (0.92–1.59)	1.33 (0.58–3.04)	1.20 (0.89–1.60)	1.12 (0.83–1.49)	1.21 (0.53–2.78)	1.11 (0.81–1.50)

a Adjusted for age and gender.

b Adjusted additionally for education, smoking status and obesity.

Each respondent diagnosed with a particular disorder may be also diagnosed with the other disorders.

**Table 2.** HRs (95% CIs) for all-cause mortality associated with single DIS-based mental disorders; SCS, 1992−2011

				Hazard ratio (95% CI)					
	No. (%) diagnosed			Adjusted for age			All covariates^b^		
	All (*n* = 1397)	Women (*n* = 759)	Men (*n* = 638)	All^a^	Women	Men	All^a^	Women	Men
Depression only	13 (0.93%)	12 (1.58%)	1 (0.16%)	1.81 (0.58–5.69)	1.83 (0.58–5.77)	n/a	1.79 (0.57–5.64)	1.79 (0.57–5.65)	n/a
Dysthymia only	8 (0.57%)	6 (0.79%)	2 (0.31%)	1.04 (0.33–3.27)	0.72 (0.18–2.90)	5.50 (0.75–40.16)	1.09 (0.35–3.42)	0.75 (0.19–3.04)	5.72 (0.78–42.02)
Panic disorder only	3 (0.21%)	1 (0.13%)	2 (0.31%)	7.4 (2.34–23.43)	23.37 (3.16–172.94)	5.75 (1.41–23.54)	6.62 (2.07–21.21)	27.83 (3.74–206.98)	4.95 (1.2–20.47)
Gad only	130 (9.31%)	87 (11.46%)	43 (6.74%)	1.02 (0.74–1.41)	0.91 (0.59–1.38)	1.21 (0.74–1.99)	0.98 (0.71–1.37)	0.88 (0.57–1.34)	1.19 (0.70–2.02)
Alcohol use disorder only	211 (15.11%)	23 (3.03%)	188 (29.47%)	1.10 (0.8–1.52)	1.31 (0.42–4.15)	1.71 (1.07–2.74)	1.02 (0.73–1.43)	1.16 (0.36–3.67)	1.04 (0.73–1.48)

a Adjusted for age and gender.

b Adjusted additionally for education, smoking status and obesity.

In order to assess the degree to which lifetime comorbidity increased mortality risk, a final set of regressions was conducted. Comorbidity was defined as subjects meeting lifetime criteria for two conditions at study baseline in 1992. For each pairwise combination of disorders, participants with both disorders (comorbid group) were compared with those with neither of the two (reference group) (Table 3). Lastly, all models above were further adjusted for education, smoking habits and obesity.

**Table 3.** HRs (95% CIs) for all-cause mortality associated with co-diagnosis of DIS-based mental disorders; SCS, 1992−2011

				Hazard Ratio (95% CI)					
	No. (%) diagnosed			Adjusted for age			All covariates^b^		
	All (*n* = 1397)	Women (*n* = 759)	Men (*n* = 638)	All^a^	Women	Men	All^a^	Women	Men
Depression and dysthymia	49 (3.51%)	38 (5.01%)	11 (1.72%)	1.28 (0.74–2.19)	0.88 (0.43–1.78)	3.01 (1.33–6.83)	1.18 (0.68–2.02)	0.83 (0.41–1.69)	2.56 (1.12–5.89)
Depression and panic	18 (1.29%)	13 (1.71%)	5 (0.78%)	1.95 (0.72–5.28)	1.95 (0.48–7.94)	1.97 (0.49–7.98)	1.87 (0.69–5.05)	1.82 (0.45–7.44)	1.94 (0.48–7.85)
Depression and GAD	81 (5.8%)	56 (7.38%)	25 (3.92%)	1.23 (0.76–1.99)	0.91 (0.47,1.79)	1.84 (0.94–3.61)	1.19 (0.74–1.93)	0.90 (0.46–1.76)	1.71 (0.87–3.38)
Depression and alcohol	34 (2.44%)	15 (1.98%)	19 (2.98%)	2.47 (1.30–4.71)	1.74 (0.43–7.07)	2.79 (1.35–5.75)	2.20 (1.15–4.22)	1.61 (0.40–6.60)	2.45 (1.18–5.09)
Dysthymia and panic	19 (1.36%)	15 (1.98%)	4 (0.63%)	2.14 (0.95–4.83)	1.71 (0.63–4.63)	4.12 (1.01–16.77)	1.91 (0.84–4.35)	1.53 (0.56–4.16)	3.72 (0.91–15.23)
Dysthymia and GAD	68 (4.87%)	49 (6.46%)	19 (2.98%)	1.27 (0.82–1.97)	1.09 (0.63–1.88)	1.71 (0.84–3.40)	1.19 (0.77–1.84)	1.05 (0.61–1.82)	1.48 (0.72–3.06)
Dysthymia and alcohol	30 (2.15%)	16 (2.11%)	14 (2.19%)	1.87 (0.96–3.66)	2.29 (0.72–7.25)	1.74 (0.77–3.95)	1.62 (0.82–3.21)	2.23 (0.70–7.07)	1.45 (0.63–3.34)
Panic and GAD	28 (2.00%)	22 (2.90%)	6 (0.94%)	1.95 (0.96–3.96)	1.99 (0.88–4.53)	1.74 (0.43–7.06)	1.83 (0.90–3.74)	1.84 (0.81–4.20)	1.74 (0.43–7.08)
Panic and alcohol	7 (0.50%)	3 (0.40%)	4 (0.63%)	4.03 (1.27–12.79)	7.57 (1.04–55.31)	3.27 (0.8–13.38)	3.80 (1.19–12.16)	6.74 (0.91–49.65)	3.12 (0.75–12.87)
GAD and alcohol	73 (5.23%)	22 (2.90%)	51 (7.99%)	1.47 (0.91–2.37)	2.42 (0.75–7.83)	1.4 (0.83–2.36)	1.31 (0.80–2.13)	2.10 (0.65–6.83)	1.24 (0.73–2.12)

a Adjusted for age, gender and the presence of both diagnoses included.

b Adjusted additionally for education, smoking status and obesity.

## RESULTS

A total of 1397 respondents were followed-up with respect to mortality for 19 years. In total, 442 deaths (225 among women and 217 among men) were observed during the follow-up.

Prevalence of lifetime DIS disorders in the 1992 sample ranged from 21.6% for alcohol use disorder to 2.5% for panic disorders. Women had at least two times higher prevalence of all the disorders except for alcohol abuse or dependence, which was more than five times more prevalent among men than among women (Table 1).

Despite the higher prevalence of depression and panic disorder among women, mortality risk associated with these two conditions was significantly increased only in men. The elevated risk persisted even after adjusting for education, smoking and obesity: among men, lifetime history of depression (with or without additional comorbidities) was associated with twice the risk of mortality (HR 2.08; 95% CI 1.15–3.75), and lifetime history of panic disorder was associated with nearly three times the risk of mortality (HR 2.87; 95% CI 1.17–7.07). In sensitivity analyses, those with panic disorder alone had 6.5 times the risk of mortality compared with participants with no disorder (HR 6.62, 95% CI 2.07–21.21).

The highest prevalence of comorbidity between two disorders was observed for GAD and depression (5.8%), followed by alcohol abuse or dependence and GAD (5.2%), GAD and dysthymia (4.9%), and depression and dysthymia (3.5%). Men were much more likely than women to meet diagnostic criteria for both alcohol use disorder and GAD (8 *v.* 2.9%), whereas women more often met criteria for all other combinations of disorders (Table 3).

Again, higher prevalence of comorbidity among women did not translate to higher risk of mortality associated with these diagnoses. After adjusting for potential confounders, men with depression and dysthymia had 2.56 (95% CI 1.12–5.89) times the mortality risk than those with neither diagnosis, and those diagnosed with depression and alcohol use disorder had 2.45 (95% CI 1.18–5.10) times the risk of death than those without the two diagnoses. In the combined sample (males and females), alcohol use disorder seemed to increase risk of mortality among those also meeting criteria for depression, dysthymia and GAD: HRs for depression, dysthymia and GAD with additional alcohol use disorder were 2.20 (95% CI 1.15–4.22), 1.62 (95% CI 0.82–3.21) and 1.31 (95% CI 0.80–2.13), respectively, while HRs for depression, dysthymia and GAD alone were 1.79 (95% CI 0.57–5.64), 1.09 (95% CI 0.35–3.42) and 0.98 (95% CI 0.71–1.37) (Tables 2 and 3).

## DISCUSSION

Results from this large community-based cohort study suggest that lifetime comorbidity between common mental disorders is both common and deleterious with respect to mortality.

Prevalence estimates suggested that the experience of more than one common mental disorder over the lifetime is the rule, rather than its exception. With the exception of alcohol use disorders, participants far more often met lifetime criteria for two or more disorders than a single diagnosis. The most common lifetime comorbidities encountered were between mood disorders (major depression; dysthymia) and GAD. Those who had experienced depression, dysthymia and GAD were also highly likely to meet lifetime criteria for alcohol use disorders. These findings are in agreement with an abundance of previous evidence showing a strong relationship between depression and anxiety (Dobson, 1985; eds Maser and Cloninger, 1990; Lovibond and Lovibond, 1995), and between mood and anxiety disorders and alcohol use disorders (Lai *et al*., 2015).

### Mood disorders and mortality

Depression has been consistently associated with excess mortality in community samples (Cuijpers *et al*., 2014*a*), and some literature suggests that men may bear a disproportionate burden of this risk (Cuijpers *et al*., 2014*b*; Gilman *et al*., 2017), though our prior investigation across the six-decade span of the Stirling County Study using a different diagnostic interview for depression suggests that an excess mortality risk among females with depression emerged in the later years of the study (6). Here, mortality risk according to the DIS was doubled among men meeting lifetime criteria for major depressive episode.

The superimposition of major depression on chronic dysthymia is often referred to in the literature as ‘double depression’ (Keller and Shapiro, 1982). It has been noted that double depression is present in a majority of cases of dysthymia (Rhebergen *et al*., 2009). Results from the present study supported this assertion – a majority of individuals meeting criteria for dysthymia also met criteria for major depression.

Double depression has been associated with worse functional outcomes than major depression alone (Rhebergen *et al*., 2009). In line with these findings, in the present study, co-morbidity between major depression and dysthymia was associated with an increased risk of mortality among men. Small sample sizes precluded direct comparison between those with ‘double depression’ and major depression alone – only one male met criteria for depression in the absence of other disorders. Nonetheless, these results suggest that the combination of both chronic and episodic depressive symptoms may place men at heightened risk of death.

Mechanisms linking depression and mortality are not fully understood, though recent evidence points to increased inflammation among depressed patients (Russ *et al*., 2012), as well as changes in cardiovascular functioning (Larson *et al*., 2001), as potential explanatory factors. Depression and dysthymia are additionally associated with poor diet quality, physical inactivity, smoking and alcohol use, which may also explain increased mortality risk (Dierker *et al*., 2002; Holahan *et al*., 2003; Whooley *et al*., 2008).

### Anxiety disorders and mortality

With respect to anxiety, our results indicated that consistent with earlier results of the Stirling County study (Murphy *et al*., 1987), GAD on its own, or in combination with other diagnoses, was not a significant predictor of mortality. In fact, the experience of GAD with another lifetime diagnosis seemed to be associated with lower risk of mortality than that for single disorders with no GAD, although small sample sizes precluded our ability to conduct direct statistical comparisons. Though mechanisms are not well understood, it has been suggested that anxiety may confer a protective effect on mortality. Anxiety seems to serve an evolutionary function as a threat-detection mechanism (Bateson *et al*., 2011), and some have suggested it may reduce mortality through the increased use of healthcare (Simon, 1992).

In contrast, a lifetime diagnosis of panic disorder alone was associated with substantially higher risk of mortality in the full sample – individuals with panic disorder had over six times higher mortality risk than those without. However, as only three individuals met criteria for this diagnosis in isolation, results should be interpreted with caution. Nonetheless, when all individuals with panic (irrespective of other diagnoses) were considered, the risk of death was 2.34 times higher than for participants without panic. Few studies have examined the association between panic disorder and mortality; notwithstanding, this finding is consistent with results from a small US study reporting increased mortality risk among former psychiatric inpatients with panic disorder (Coryell *et al*., 1982). Panic disorder has been associated with elevated levels of certain pro-inflammatory cytokines (Hoge *et al*., 2009) which may play a role in the development of cardiovascular disease.

Though three times as many women as men met criteria for panic disorder, the association with mortality in the present study was strongest among males. This result is consistent with a study from the Lundby cohort, which reported an increased mortality risk among older males, but not females, with panic disorder (Gräsbeck *et al*., 1996).

### Alcohol use disorders

In this study, alcohol use disorders were not, by themselves, associated with excess mortality. However, individuals meeting lifetime criteria for an alcohol use disorder who had also experienced major depression or panic disorder in their lifetime were at significantly elevated risk of mortality. The combination of alcohol use disorders with dysthymia and GAD also seemed to confer increased risk – though these differences were not statistically significant. These results are consistent with previous research suggesting that comorbidity of mood and anxiety disorders with alcohol use disorders is associated with poorer prognosis, including increased risk of impairment, relapse and suicide (Merikangas *et al*., 1998; Smith and Randall, 2012).

Alcohol misuse is common among individuals with mood and anxiety disorders (Lai *et al*., 2015), and may reflect self-medication – that is, the use of alcohol to relieve unpleasant symptoms of depression and anxiety (Bolton *et al*., 2006, 2009). Self-medication of mood and anxiety disorders has been associated with deleterious outcomes, including greater likelihood of psychiatric comorbidities (Bolton *et al*., 2009), and higher rates of suicidal ideation and attempt (Bolton *et al*., 2006). However, alcohol dependence has also been found to be a risk factor for the subsequent development of depression (Gilman and Abraham, 2001), suggesting that the association between alcohol use disorders and other psychiatric disorders are complex and bi-directional.

### Strengths and limitations

Results of this study should be interpreted in light of certain limitations. First, the assessment of mental disorder was conducted at a single time point and included retrospective reports of symptoms over the lifetime. It is likely that some cases remitted during the follow-up period, while others persisted or relapsed. Studies including multiple measurements of mental disorder over time would be better placed to assess the importance of chronicity for outcomes, including mortality. Second, as mentioned, sample sizes for single disorders were very small – for example, only one male met diagnostic criteria for major depression in the absence of other disorders. To draw stronger conclusions about the impact of comorbidities over and above unique diagnoses, larger samples of individuals with each combination of disorders are required.

Notwithstanding, we believe that the use of a community-based random sample is a strength of the current study. Further strengths of the study include its long follow-up (19 years of mortality follow-up) and structured assessments of mental disorders. In addition, few other studies have assessed the mortality burden of comorbid mental illness separately for males and females.

Taken together, results of this study suggest that the cumulative experience of more than one mood, anxiety or alcohol use disorder is extremely common. Comorbid mental disorders were associated with increased risk of mortality, most strikingly among men. Existing literature linking mental disorder with mortality risk has tended to focus on single disorders – these results suggest a shift towards recognising the significant heterogeneity in the presentation of common mental disorders, particularly the importance of considering symptoms of multiple psychiatric conditions over the lifetime.

## Availability of data and materials

Data for the Stirling County Study are available through the Harvard MIT data centre. Contact Dr J M Murphy for access approval.
